# In Vivo Quantification of Surfactin Nonribosomal Peptide Synthetase Complexes in *Bacillus subtilis*

**DOI:** 10.3390/microorganisms12112381

**Published:** 2024-11-20

**Authors:** Maliheh Vahidinasab, Lisa Thewes, Bahar Abrishamchi, Lars Lilge, Susanne Reiße, Elvio Henrique Benatto Perino, Rudolf Hausmann

**Affiliations:** 1 Department of Bioprocess Engineering (150k), Institute of Food Science and Biotechnology, University of Hohenheim, Fruwirthstrasse 12, 70599 Stuttgart, Germany; lisa.thewes@uni-hohenheim.de (L.T.); bahar.abrishamchi@uni-hohenheim.de (B.A.); lars.lilge@uni-hohenheim.de (L.L.); eperino@uni-hohenheim.de (E.H.B.P.); 2 Imaging Unit, Core Facility of Hohenheim, Emil-Wolff-Strasse 12, 70599 Stuttgart, Germany; susanne.reisse@uni-hohenheim.de

**Keywords:** non-ribosomal peptide synthetases, NRPS, in vivo quantification, GFP, *Bacillus subtilis*, surfactin, lipopeptide, biosurfactant

## Abstract

Surfactin, a potent biosurfactant produced by *Bacillus subtilis*, is synthesized using a non-ribosomal peptide synthetase (NRPS) encoded by the *srfAA-AD* operon. Despite its association with quorum sensing via the ComX pheromone, the dynamic behavior and in vivo quantification of the NRPS complex remain underexplored. This study established an in vivo quantification system using fluorescence labeling to monitor the availability of surfactin-forming NRPS subunits (SrfAA, SrfAB, SrfAC, and SrfAD) during bioprocesses. Four *Bacillus subtilis* sensor strains were constructed by fusing these subunits with the *megfp* gene, resulting in strains BMV25, BMV26, BMV27, and BMV28. These strains displayed growth and surfactin productivity similar to those of the parental strain, BMV9. Fluorescence signals indicated varying NRPS availability, with BMV27 showing the highest and BMV25 showing the lowest relative fluorescence units (RFUs). RFUs were converted to the relative number of NRPS molecules using open-source FPCountR package. During bioprocesses, NRPS availability peaked at the end of the exponential growth phase and declined in the stationary phase, suggesting reduced NRPS productivity under nutrient-limited conditions and potential post-translational regulation. This study provides a quantitative framework for monitoring NRPS dynamics in vivo, offering insights into optimizing surfactin production. The established sensor strains and quantification system enable the real-time monitoring of NRPS availability, aiding bioprocess optimization for industrial applications of surfactin and potentially other non-ribosomal peptides.

## 1. Introduction

Fluorescence reporters are commonly used for quantitatively studying the behavior of natural and engineered target proteins [1]. Therefore, a wide range of fluorescent protein tags have been applied, including fluorescence proteins with different spectral properties [2]. In the case of the green fluorescent protein (GFP), fluorescent tags have been applied for analyzing the localization, structure, and dynamics of macromolecules in living cells [3,4]. Consequently, the localization of proteins involved in sporulation and cell division was determined in the target organism *Bacillus subtilis* [5,6,7]. In addition, dynamics of proteins could be visualized and determined in real time, such as the replication machinery in *B. subtilis* [8]. Since the self-assembled domain structure of GFP reduces the potential for interference with protein fluorescence by fused proteins and, vice versa, the activities of the proteins to which it is fused, GFP tags are promising analytical tools for the analyses of enzymatic performances of target proteins [3,9,10].

Exemplarily, non-ribosomal peptide synthetases (NRPSs) are large multifunctional enzyme complexes responsible for the biosynthesis of non-ribosomally produced secondary metabolites in various organisms, including bacteria and fungi. These enzymes incorporate a diverse range of amino acids, including unusual ones, leading to a wide variety of natural structures and bioactivities, such as antibacterial, antiviral, and especially antifungal properties [11,12,13]. Consequently, many metabolites derived from NRPSs are interesting for biotechnological applications [14,15,16,17].

*Bacillus* species are the leading producers of lipopeptides, known for their potent biological properties, such as antifungal activity, surpassing other lipopeptide-producing bacteria in terms of quantity [18]. While lipopeptides from *Pseudomonas* or *Streptomyces* species typically yield only a few milligrams per liter, certain wild-type *Bacillus* strains can produce approximately one gram per liter [19,20]. Although these levels are not yet ideal for agricultural bio-fungicide applications, they indicate significant potential for improvement. Consequently, research focused on optimizing *Bacillus* lipopeptides is growing rapidly.

Surfactin, a cyclic lipopeptide produced by an NRPS encoded by the *srfAA-AD* operon in *Bacillus* spp., is one of the most effective biosurfactants discovered to date, exhibiting a broad spectrum of biological activities [20,21,22]. Surfactin’s unique structure significantly enhances its interactions, especially in surface tension reduction, antimicrobial activity, and interactions with cell membranes [23]. Surfactin is composed of a cyclic peptide moiety (l-glutamate, l-leucine, d-leucine, l-valine, l-aspartate, d-leucine, and l-leucine) which is cyclically linked to a β-hydroxy fatty acid that could be linear or have iso or anteiso branches with 12 to 17 carbons [24]. To guarantee the structural organization, the surfactin-forming NRPS coordinates the assembly of the surfactin molecule through a modular and highly specific enzyme-mediated process [15]. NRPSs function as a multi-enzyme complex that operates like a molecular assembly line, with each module responsible for modifying a specific amino acid in the growing peptide chain. This process is distinct from ribosomal peptide synthesis as it allows the incorporation of non-standard amino acids and the production of cyclic peptides. However, the NRPS enzyme complex requires a post-translational activation for functionalization. Accordingly, Sfp catalyzes the phosphopantetheinylation of specific serine residues in the seven peptidyl carrier protein domains of the first three surfactin-forming NRPS subunits (SrfAA-SrfAB-SrfAC) [25,26]. Despite the key role of this large enzyme complex in the bioproduction of surfactin, the dynamic behavior of NRPS complexes in vivo remains inadequately understood. This lack of knowledge prevents the accurate estimation of the number of NRPS molecules per cell and the ratio between the number of NRPS and the moles of surfactin produced during cultivations of the producer bacteria in an appropriate medium [27].

This study presents the establishment of an in vivo quantification system for surfactin-forming NRPS complexes, aiming to estimate the production rate of NRPS complexes during bioprocesses. Therefore, a fusion protein system was developed using functional NRPS subunits coupled with the fluorescence protein mEGFP. In this way, quantitative insight into NRPS complexes could be achieved.

## 2. Materials and Methods

### 2.1. Construction of B. subtilis Sensor Strains

The plasmids and strains used in this study are listed in Table S2 in Supplementary File S1. All plasmids were generated from the initial plasmid pJOE6743.1 [28] using a Gibson Assembly protocol as described by the manufacturer (New England Biolabs, Frankfurt am Main, Germany). In this way, the flanking regions of about 1000 bp for homologous chromosomal integration were linked with the *megfp* gene. The Gibson Assembly reaction mixture was used for transformation in *E. coli* strain DH5α.

Afterward, constructed plasmids pMAV22 (*srfAA*-*megfp*), pMAV23 (*srfAB*-*megfp*), pMAV24 (*srfAC*-*megfp*), and pMAV25 (*srfAD*-*megfp*) were applied for transformation using the surfactin-producing *B. subtilis* target strain BMV9, enabling a subsequent mannose counterselection for markerless mutant strain construction [29]. For mutant strain selection, the following antibiotics were used: ampicillin (100 µg/mL) and spectinomycin (100 µg/mL).

### 2.2. Cultivation Media

Main cultivations for surfactin bioproduction were performed in a chemically defined mineral salt medium, as previously described in [30]. The preliminary pre-culture cultivation was prepared in LB medium, as described in [31]. The prepared pre-cultures were used to inoculate the main cultivation in a 96-well plate or in a shake flask, as described in the following sections.

### 2.3. Real-Time Monitoring of Cell Growth and Fluorescence Signals in Plate Reader

The constructed *B. subtilis* mutant strains BMV25–BMV28, as well as the parental strain BMV9, were cultivated in a volume of 200 µL with a starting optical density of 0.1 in a 96-well plate. The cultures were performed in triplicate and incubated for 12 h at 37 °C in a fluorescence plate reader (FLUOstar Omega, BMG LABTECH GmbH, Ortenberg, Germany). Optical density at 600 nm (OD_600_) and the fluorescence signal (excitation at 485 nm, emission at 520 nm) were monitored every 10 min in each well.

### 2.4. Shake Flask Cultivations and Determination of Living Cells

Bacterial cells were cultivated for 33 h in 1 L baffled shake flasks using a filling volume of 10% for the cultivation medium. All cultivations were carried out in biological triplicate and were performed at 37 °C, with 0.4 g, and at 120 rpm in an incubation shaker (Innova 44^®^R, Eppendorf AG, Hamburg, Germany). Samples were taken regularly for further analyses of surfactin production and fluorescence signal measurement. The number of living cells per volume was determined as described in [32].

### 2.5. Surfactin Analysis

The concentration of surfactin produced during the shake flask cultivation was measured as previously described in [33]. In brief, a volume of 2 mL cell-free supernatant was extracted three times with chloroform/methanol (2:1). The pooled solvent layers were dried using a rotary evaporator at 10 mbar and 40 °C. Dried samples were resolved in 2 mL methanol and applied in 6 mm bands on a silica HPTLC plate. A mixture of chloroform/methanol/water (65:25:4) was used as a mobile phase, and a migration distance of 60 mm was applied. Surfactin standard from Sigma Aldrich was used for quantification.

### 2.6. Fluorescence Signal Measurement

The fluorescence signals emitted by the mEGFP protein were measured using a fluorescence plate reader (FLUOstar Omega, BMG LABTECH GmbH, Ortenberg, Germany). During shake flask cultivation, at each sampling time point after 9 h, 100 µL samples were transferred in triplicate to a 96-well plate, and the fluorescence signal was measured with the following settings. For the kinetic experiment, the fluorescence signal was measured directly in each well during cultivation. A GFP filter set was used with excitation at 485 nm (12 nm width) and emission at 520 nm. The fluorescence signals of mEGFP were measured with a gain of 1000 and 21 flashes using bottom optics. Each well was scanned as an orbital average with a diameter of 4 mm. The measured signals were corrected for background fluorescence caused by autofluorescence according to [34]. For this purpose, the background fluorescence intensity (FIRef) was measured using the parental *B. subtilis* strain BMV9 as a negative reference exhibiting no fluorescent protein. The corrected fluorescence intensity (FI_corrected_) was calculated with the following equation:

FI_corrected_ [-] = FI_uncorrected_ − (A_600nm,corrected_/A_600nm, Ref_) × FI_Ref_ (1)

The relative fluorescence unit RFU is given by the FI_corrected_ per optical density determined with the corrected absorption signal at 600 nm A_600nm,corrected_:

RFU [-] = FI_corrected_/Cell number (2)

### 2.7. Expression and Purification of mEGFP

Plasmid pET-28a was used for the construction of pMAV35, encoding a his-tagged *megfp* gene expressed by a constitutively active T7 promoter. After the transformation of pMAV35 in *E. coli* BL21(DE3) Gold and cultivation in LB medium with ampicillin as the selection marker for 24 h until OD_600_ of 0.8, *megfp* gene expression was induced through the addition of 1 mM (*w*/*v*) IPTG at 37 °C and 120 rpm. Subsequently, the bacterial cells were harvested by centrifugation at 4700 rpm for 10 min and 4 °C, and the cell pellet was used for mechanical cell disruption with a high-pressure homogenizer (SPX, Charlotte, NC, USA). Therefore, the cell pellets were resuspended in binding buffer (20 mM sodium phosphate, 500 mM NaCl, 30 mM imidazole; pH 7.4). The cells were disrupted at approximately 1400 bar in 4 cycles. The resulting cell suspension was centrifuged for 12,000 rpm for 30 min at 4 °C to separate the insoluble and the soluble protein fraction. Since mEGFP is a soluble protein, only the supernatant was used for further purification.

Next, the purification of mEGFP was performed by immobilized metal ion affinity chromatography (IMAC) using an automated chromatography system (ÄKTA^TM^ Start, Cytiva Europe GmbH, Freiburg, Germany) according to the manufacturer’s instructions. In detail, his-tagged mEGFP was purified using a HisTrapFM HP column prepacked with pre-charged Ni SepharoseTM using a column volume (CV) of 5 mL. Before purification, the system was washed with 5 CV of water and equilibrated with 5 CV of binding buffer. Subsequently, the sample was applied to the column, washed with binding buffer and eluted using a one-step gradient (100%) with elution buffer (20 mM sodium phosphate, 500 mM NaCl, 500 mM imidazole; pH 7.4). The elution fractions were pooled, and a subsequent desalting step was performed to remove the imidazole from the purified protein by repeating the purification described above with a HiTrapTM desalting column. Therefore, a desalting buffer (20 mM sodium phosphate, 500 mM NaCl; pH 7.4) was applied. The total protein concentration of the purified samples was determined with the Bradford method [35].

### 2.8. Calibration of mEGFP Activity Using FPCountR

The calibration of fluorescence data sets obtained using the fluorescence plate reader FLUOstar (BMG Labtech, Ortenberg, Germany) was performed as previously described in detail in [36] using the Github-implemented FPCountR package and the provided protocol. This open source R package provided all relevant functions for calculating a conversion factor that relates the relative fluorescence signal measured in the fluorescence plate reader with the number of fluorescence protein molecules. To determine the protein concentration, the molecular weight (get_mw()) of mEGFP was calculated, and the extinction coefficient (EC) was obtained from the FPbase database. These values were used to calculate the ECmax mass extinction coefficient, which was then used for the conversion of the protein concentration using three different correction methods: none, baseline, and light scatter correction. All correction methods were compared within the function, but scatter normalization was chosen for further analysis, using the scatter_ratio between A390nm and the ECmax wavelength.

The function get_conc_ECmax() produced linear models fitted for each correction method between dilution and predicted protein concentrations. In a final step, generate_cfs() was used to calculate a conversion factor (cf) based on a model that relates protein concentrations to relative fluorescence units. Consequently, the calculated cf was used to convert corrected relative fluorescence units (RFUs or FI_corrected_) to the number of protein molecules equivalent with mEGFP (MEFP) per bacterial cell:

mEGFP molecules/Cell = RFU/*cf*_mEGFP,FLUOstar,filter:ex485/12nm,em520nm,gain1000_ (3)

where *cf* is specific to the fluorescent protein, the plate reader, filter set, and gain.

### 2.9. Microscopy

For visualizing cells from exponentially growing cultures, bacteria were grown in mineral salt medium, and samples were taken after 16 h. The cells were washed twice in phosphate-buffered saline (PBS, 137 mM NaCl, 2.7 mM KCl, 10 mM Na_2_HPO_4_, and 1.8 mM KH_2_PO_4_ in 1 L DI water; pH 7.4), and cell fixation was carried out using 4% (*v*/*v*) paraformaldehyde in PBS for 30 min. After fixation, cells were washed twice in PBS and mounted on 1% (*w*/*v*) agarose pads [37]. Final microscopic images of the fluorescent cells were captured using the Zeiss LSM 900 microscope (Zeiss, Jena, Germany), which was equipped with Airyscan 2.

## 3. Results

### 3.1. Online Monitoring of Surfactin-Producing B. subtilis Sensor Strains with NRPS Subunits Labeled with mGFP-Tags

**Figure 1:**
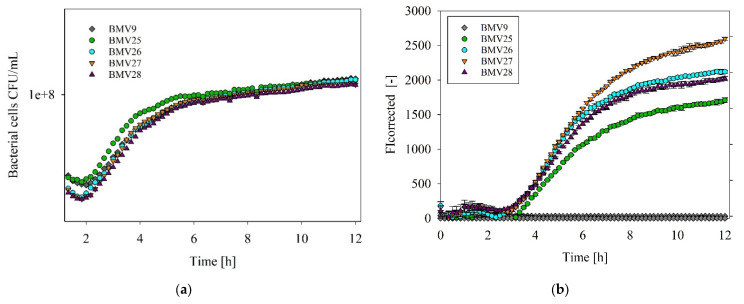
Online monitoring of cell growth and fluorescence intensity (FI) of *B. subtilis* sensor strains. Optical density (**a**) and relative fluorescence intensity (**b**) were determined for the constructed *B. subtilis* mutant strains encoding *srfA* genes C-terminally fused with a *megfp* protein tag over a 12 h period in 96-well plate cultivations. Hence, the parental control strain BMV9 (diamond) and the sensor strains BMV25 (*srfAA*-*megfp*, green cycle), BMV26 (*srfAB*-*megfp*, cyan cycle), BMV27 (*srfAC*-*megfp*, inverted orange triangle), and BMV28 (*srfAD*-*megfp*, violet triangle) were cultured in biological triplicates.

With the use of mannose-counterselection for the construction of markerless mutant strains, the parental *B. subtilis* strain BMV9 was used for the individual chromosomal C-terminal fusion of the genes *srfAA*, *srfAB*, *srfAC*, and *srfAD*, encoding the surfactin-forming NRPS enzyme complex with an mEGFP gene. Subsequently, the constructed *B. subtilis* mutant strains BMV25 (*srfAA*-*megfp*), BMV26 (*srfAB*-*megfp*), BMV27 (*srfAC*-*megfp*), and BMV28 (*srfAD*-*megfp*) were applied in 96-well plate cultivations for monitoring their cell growth and fluorescence signals, which were representative of NRPS availability (Figure 1).

In this context, all mutant strains revealed cell growth patterns comparable to the parental strain BMV9 over a 12 h period of cultivation, suggesting no detectable impact of the mEGFP protein tag on bacterial physiology (Figure 1a). In contrast, the fluorescence signals measured during the cultivation process showed clear variations among the *B. subtilis* sensor strains. While no fluorescence was determined for the parental strain BMV9 as a negative control strain, the strains BMV27 (*srfAC*-*megfp*) and BMV25 (*srfAA*-*megfp*) revealed the highest (~2597) and lowest (~1707) fluorescence intensity values, indicating variations in the activity of the mEGFP proteins fused to the SrfA subunits. Overall, relative fluorescence intensity values determined for all sensor strains reached a plateau during the cultivation process, suggesting an almost constant NRPS availability after entering the stationary phase (Figure 1b).

### 3.2. Visual Distribution of Surfactin-Forming NRPS Subunits in B. subtilis

**Figure 2:**
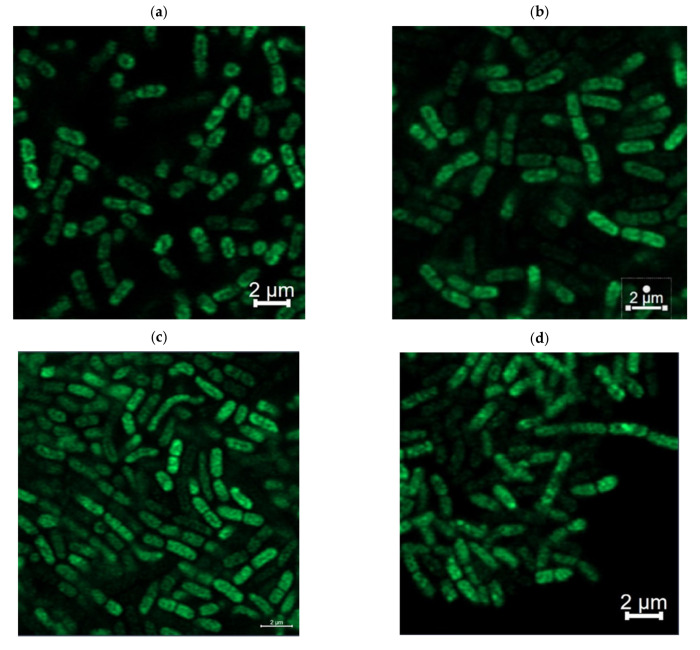
Fluorescence microscopic image of bacterial strains cultivated in mineral salt medium until the middle of the exponential phase. *B. subtilis* BMV25 (*srfAA-megfp*) (**a**), *B. subtilis* BMV26 (*srfAB-megfp*) (**b**), *B. subtilis* BMV27 (*srfAC-megfp*) (**c**), and *B. subtilis* BMV28 (*srfAD-megfp*) (**d**) showing the localization of surfactin-forming NRPS subunits with C-terminal-fused mEGFP protein.

In the next step, the *B. subtilis* sensor strains were utilized for fluorescence microscopy to investigate the distribution of surfactin-forming non-ribosomal peptide synthetases (NRPSs). As shown in Figure 2, the fluorescence signals of all four constructed sensor strains BMV25–BMV28 were non-homogenously distributed over the cells. Accordingly, surfactin biosynthesis seems to be associated with bioproduction hotspots associated especially along the cell periphery, confirming previous findings describing membrane localization of the surfactin-forming NRPS [38].

### 3.3. Calculation of Surfactin Productivity of B. subtilis Sensor Strains and Associated NRPS Molecules

**Figure 3:**
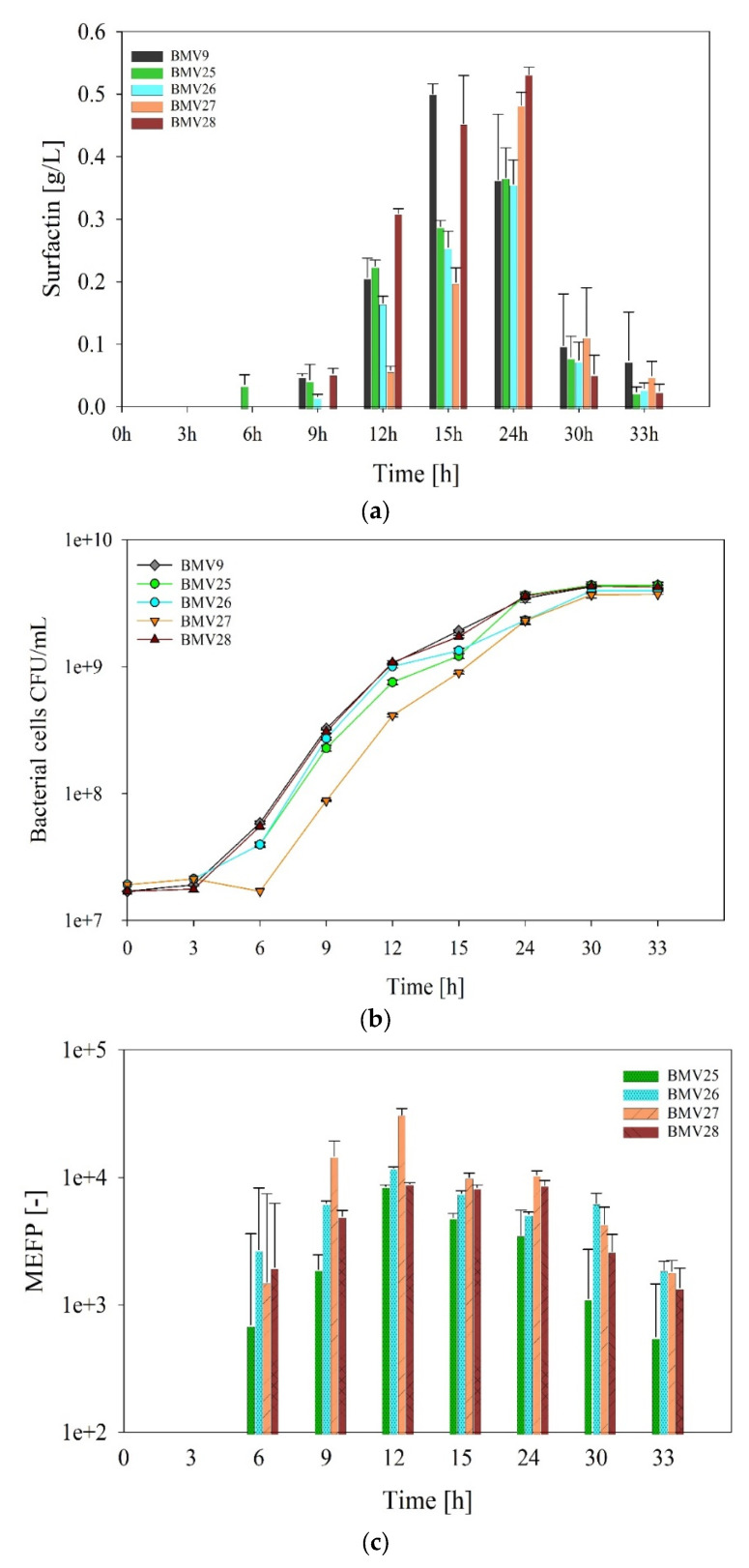
Overview of bioproduction parameters by *B. subtilis* sensor strains during the cultivation process. The parental *B. subtilis* strain BMV9 as the negative control and the sensor strains BMV25 (*srfAA*-*megfp*), BMV26 (*srfAB*-*megfp*), BMV27 (*srfAC*-*megfp*), and BMV28 (*srfAD*-*megfp*) were cultured in biological triplicates in shake flasks over a period of 33 h. During the cultivation process, surfactin (**a**), living cell numbers (**b**), and the relative number of protein molecules equivalent to mEGFP (MEFP) (**c**) were monitored.

All constructed *B. subtilis* sensor strains, BMV25 (*srfAA*-*megfp*), BMV26 (*srfAB*-*megfp*), BMV27 (*srfAC*-*megfp*), and BMV28 (*srfAD*-*megfp*), were able to produce surfactin in comparable amounts detected for their parental strain BMV9 (Figure 3a). In more detail, by growing the bacterial cells, all strains started to produce surfactin, reaching highest surfactin concentrations of approximately 0.45 and 0.5 g/L for sensor strain BMV28 (*srfAD*-*megfp*) and BMV9 reference strain at the late exponential phase. As it is shown in Figure 3b, the cells, approximately 24 h after the start of cultivation, reached high cell density, suggesting a correlation between cell density and surfactin production, likely due to the biomass-dependent activation of the *B. subtilis* quorum sensing system [39,40].

In the next step, an approximate estimate of the availability of surfactin-producing NRPS in the *B. subtilis* sensor strains was calculated. Therefore, the fluorescence signals measured for the individual *B. subtilis* sensor strains during the cultivation process were correlated with signals obtained from purified mEGFP. In this way, a calibration with FPCountR, following a protocol described previously in [36], was performed using the correlation between mEGFP protein molecules and their relative fluorescence. As a result, a calibration factor of 8.75 × 10^−10^ AU/M was calculated for the conversion of relative fluorescence units (RFUs), meaning the corrected fluorescence intensity per optical density, to mEGFP concentrations (Supplementary File S2, Figure S9). With the use of the calibration factor, RFU values were converted to the relative number of protein molecules equivalently represented by mEGFP (MEFP). The details of the mEGFP calibration results are available from Supplementary File S2. Overall, although differences in mEGFP activities might be present between the SrfA-mEGFP fusion proteins and the purified mEGFP reference protein for calibration, this approach provides an approximation of the quantity of surfactin-forming NRPS per *B. subtilis* cell (Figure 3c).

The application of the calibration allowed a prediction of the dynamic availability of the individual surfactin-forming NRPS subunits represented by the mEGFP-mediated fluorescence in the different *B. subtilis* sensor strains. At the beginning of cell growth after 6 h of cultivation, MEFP values of around 1512 to 2715 could already be calculated for all surfactin-forming NRPS subunits. In the following, an increase in the MEFP levels up to 8516 for SrfAA-mEGFP, 11,844 for SrfAB-mEGFP, 30,923 for SrfAC-mEGFP, and 8953 for SrfAD-mEGFP could be determined after 12 h of cultivation (Figure 3b,c). However, 30 h into cultivation, as the bacterial cells entered the stationary phase, these values decreased by 86%, 85%, 79%, and 70% for SrfAA, SrfAB, SrfAC, and SrfAD-mEGFP, respectively. This reduction indicates fewer surfactin-forming NRPS enzyme complexes, which aligns with the observed lower surfactin titers (Figure 3a,c).

### 3.4. Estimation of the Productivity of Surfactin-Forming NRPS Molecules

**Figure 4:**
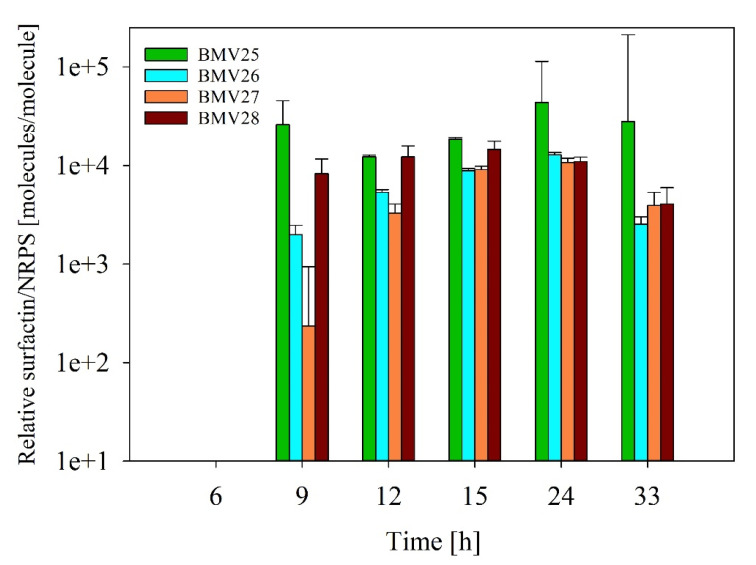
Calculation of the relative productivity of the surfactin-producing SrfA subunits. The correlation between the surfactin produced and the calculated MEFP for the *B. subtilis* sensor strains BMV25 (*srfAA*-*megfp*), BMV26 (*srfAB*-*megfp*), BMV27 (*srfAC*-*megfp*), and BMV28 (*srfAD*-*megfp*) at the beginning of the exponential growth phase until the end of cultivation after 33 h. The bar plot shows the relative bioproduction of surfactin per NRPS molecule, represented by the fluorescence of the fused mEGFP.

The productivity of NRPS enzyme complexes was estimated by calculating the relative number of NRPS subunit molecules, which were equivalent to mEGFP (MEFP). Surfactin concentrations measured during the cultivation process were compared with these calculated MEFP values. Figure 4 illustrates the relative number of surfactin molecules per NRPS molecule, starting from 9 h after the cultivation began. Notably, surfactin levels at or before 6 h were below the detection limit, so the productivity calculations were only performed for samples from 9 h onward.

In more detail, comparable productivities were calculated for all of the SrfA subunits in a range between 1270 and 14,348. Interestingly, a slight gradual decrease in NRPS productivity was observed during the exponential phase with lowest productivity values after 24 h of cultivation, which corresponds to the entry into the stationary growth phase. Accordingly, an average reduction in the SrfA productivity of 72% could be found between the mid-exponential growth phase (12 h) and the stationary phase (33 h) (Figure 4). In this way, it is reasonable to assume that the surfactin-forming NRPS complexes become less productive under limiting conditions.

## 4. Discussion

The improved bioproduction of surfactin has been addressed in a large number of studies [41,42,43]. In this context, improved availability of precursor molecules was often addressed by metabolic engineering in order to eliminate potential bottlenecks in surfactin biosynthesis [44,45,46]. Another point of optimization is the improvement of NRPS expression by changing the promoter region with the aim of optimizing the availability of the surfactin-forming enzyme complex [47,48]. Nevertheless, information on the molecular quantities of NRPS and their production performance in *B. subtilis* during established bioproduction processes has not been available yet. For this purpose, the already developed sporulation-deficient *B. subtilis* surfactin production strain BMV9 was chosen [49], which allowed for the construction of markerless sensor strains using the mannose counter-selection system [29]. Through combining the respective SrfA-NRPS enzyme subunits with a GFP tag, the NRPS availability was thus monitored over the bioprocess and the measured fluorescence provided information on the time-dependent changes in the NRPS-dependent production capacity. However, due to the significant variation in molecular weights of the surfactin synthetase subunits SrfAA, SrfAB, SrfAC, and SrfAD, the sizes of these proteins may influence GFP activity. As a result, the mEGFP expression pattern may vary slightly between different strains. Corresponding results in this work show that NRPS availability leads to a stagnation during the bioprocess with a plateau at the stationary growth phase (Figure 1). Accordingly, the NRPS enzyme quantity follows the biomass growth and the associated quorum-sensing mechanisms [39]. This indicates that the surfactin-forming biomass requires a vital and nutrient-unlimited state for maximizing NRPS availability for wild-type producers. To avoid the decline in NRPS productivity during the stationary phase, approaches such as fed-batch bioreactor cultivation, where cells stay longer in the exponential phase, are recommended. This could help maintain NRPS activity longer into the fermentation process.

In all strains, surfactin was rapidly degraded when nutrients were limited, as was observed previously in one of our last studies [50]. The exact cause of the sudden depletion of surfactin in some experiments, particularly after glucose is exhausted, remains unclear. However, it is possible that surfactin is being utilized as a nutrient source, especially under nutrient-limited conditions. *Bacillus subtilis* may metabolize the fatty acid and amino acid components of the lipopeptide, using them as sources of carbon and nitrogen.

The fluorescence-emitting nature of the mEGFP-coupled sensor construct was utilized to perform FPCountR-based calculations to determine an approximate molecular quantity of 4855. This amount was enhanced by increasing cell growth until the mid-exponential phase before it remained relatively constant over a wide range of cultivation (Figure 3). This is consistent with molecular regulatory findings of the *srfA* operon expression, which is influenced, among others, by the global transcriptional regulators CodY and AbrB (regulating cell adaptation during environmental starvation) [50,51]. In addition to the relative NRPS availability, a decreasing tendency was also calculated for the specific productivity upon entry into the stationary growth phase (Figure 4). Accordingly, a post-translational regulation also appears to exist that affects NRPS productivity and thus also influences surfactin biosynthesis after SrfA complex formation. Here, post-translational NRPS activation by the 4-phosphopantetheinyl transferase Sfp potentially plays an important role and represents a bottleneck that has not yet been considered, which plays a role particularly during the stationary phase. Previous studies have shown that improving metabolic pathways can boost surfactin production by increasing the availability of precursor molecules [52,53]. However, it is still unclear whether the limiting factor in production is the amount of these precursor molecules inside the cell or the number of NRPS enzyme complexes per cell. To better understand this, future research should focus on the balance between NRPS enzyme levels and the availability of precursor molecules needed to produce surfactin especially in large-scale processes such as in fed-batch bioreactor cultivations where the cells are longer in the exponential phase. It is noteworthy to know the maximum number of NRPS enzymes per cell in higher cell densities.

Fluorescence microscopy analyses allowed the visualization of fluorescent *B. subtilis* sensor cells (Figure 2). In this context, an unbalanced distribution of fluorescent signals could be observed with a tendency of higher signals along the cell periphery. Although a peripheral localization would support the functional role of NRPS in facilitating the synthesis and putative secretion of surfactin, the reason for the unbalanced distribution is unclear. Nevertheless, the construction of fluorescence sensor strains provides a basis for further research into the mechanisms of NRPS–membrane interactions and their implications for surfactin production.

In addition to the insights gained in this work, *B. subtilis* production strains may, in the future, serve simultaneously as biosensors. This should allow the real-time monitoring of cellular physiological states so that adaptations can be made to the bioprocess. The bioproduction of surfactin as a prominent biosurfactant with a multitude of possible application options by the microbial cell factory *B. subtilis* is intended to serve as one potential application at this point.

## 5. Conclusions and Outlook

This study provides insights into the dynamics of non-ribosomal peptide synthetase (NRPS) complexes in *Bacillus subtilis*, with a focus on surfactin production. An in vivo quantification approach, using GFP tagging, has enabled the direct observation and measurement of NRPS complexes during the bioprocess, a method that can be applied to calculate the production rate of NRPS enzyme complexes during different growth phases and conditions. Future studies will explore the in vivo production rate of the NRPS enzyme complex in other fermentation conditions, wild-type strains, and other lipopeptides such as iturin and fengycin. This knowledge should then be used to adapt cultivation parameters for the highest possible NRPS production and concentration of the produced target lipopeptide.

## Data Availability

All raw data and biological material are saved in the institute of Food Science and Biotechnology, Department of Bioprocess Engineering (150k), University of Hohenheim, Fruwirthstraße 12, Stuttgart 70599, Germany. If required, please contact the corresponding author for any detailed question.

## Supplementary Materials

The following supporting information can be downloaded at https://www.mdpi.com/article/10.3390/microorganisms12112381/s1, Supplementary File S1: A list of the oligonucleotides, bacterial strains, and plasmids used in this study; Supplementary File S2: details on mEGFP calibration using FPCountR. Refs [54,55,56] are included in the Supplementary File S1.
