# Supplementary material for: In Vivo Quantification of Surfactin Nonribosomal Peptide Synthetase Complexes in Bacillus subtilis
Source: Microorganisms. 2024 Nov 20;12(11):2381. doi: 10.3390/microorganisms12112381 (PMC11596262; doi:10.3390/microorganisms12112381)
Supplement: Supplementary file 1 [file microorganisms-12-02381-s001.zip › Supplementary File S2.pptx]

## Slide 1
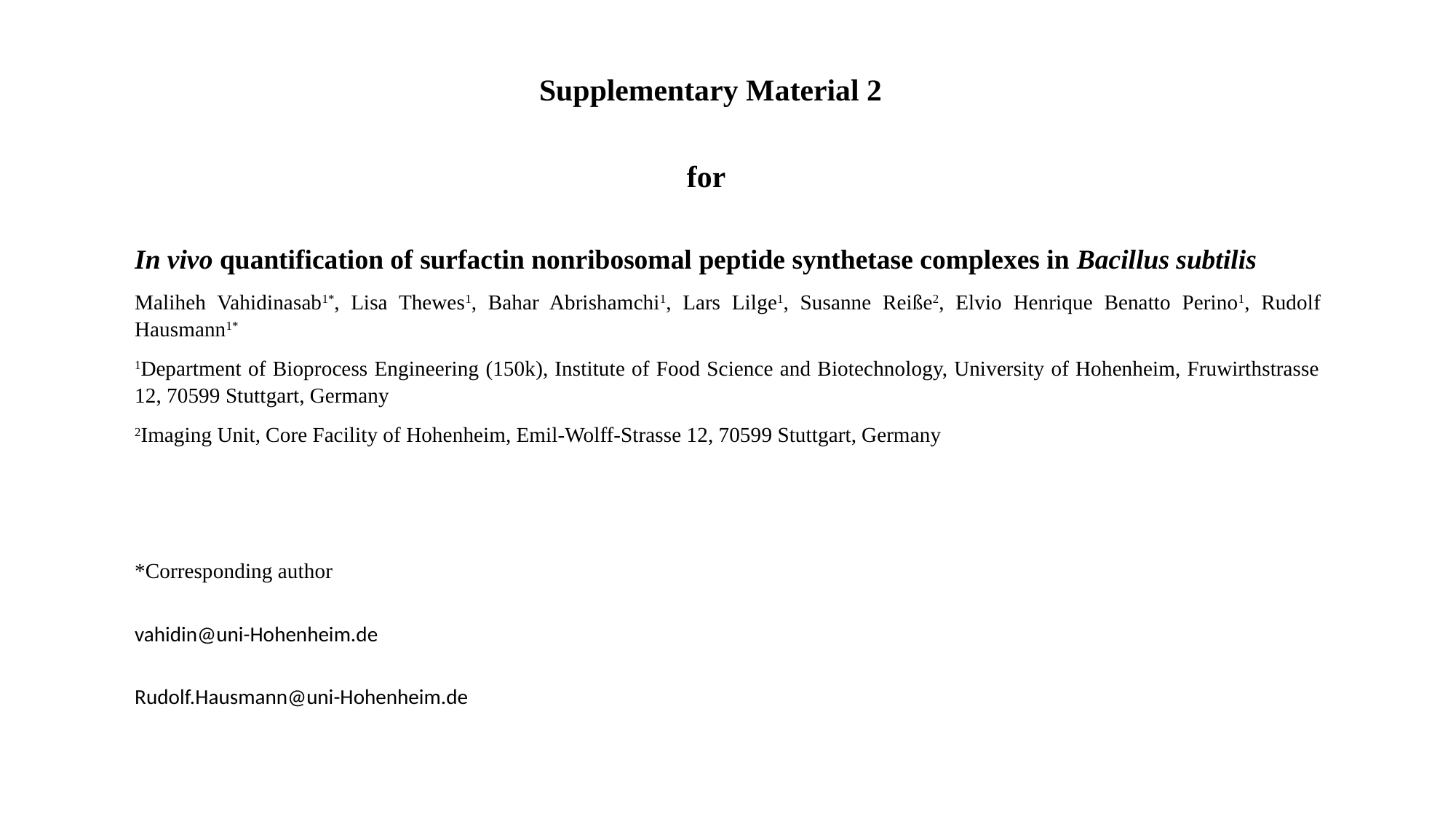

Supplementary Material 2
for
In vivo quantification of surfactin nonribosomal peptide synthetase complexes in Bacillus subtilis
Maliheh Vahidinasab1*, Lisa Thewes1, Bahar Abrishamchi1, Lars Lilge1, Susanne Reiße2, Elvio Henrique Benatto Perino1, Rudolf Hausmann1*
1Department of Bioprocess Engineering (150k), Institute of Food Science and Biotechnology, University of Hohenheim, Fruwirthstrasse 12, 70599 Stuttgart, Germany
2Imaging Unit, Core Facility of Hohenheim, Emil-Wolff-Strasse 12, 70599 Stuttgart, Germany
*Corresponding author
vahidin@uni-Hohenheim.de
Rudolf.Hausmann@uni-Hohenheim.de

## Slide 2
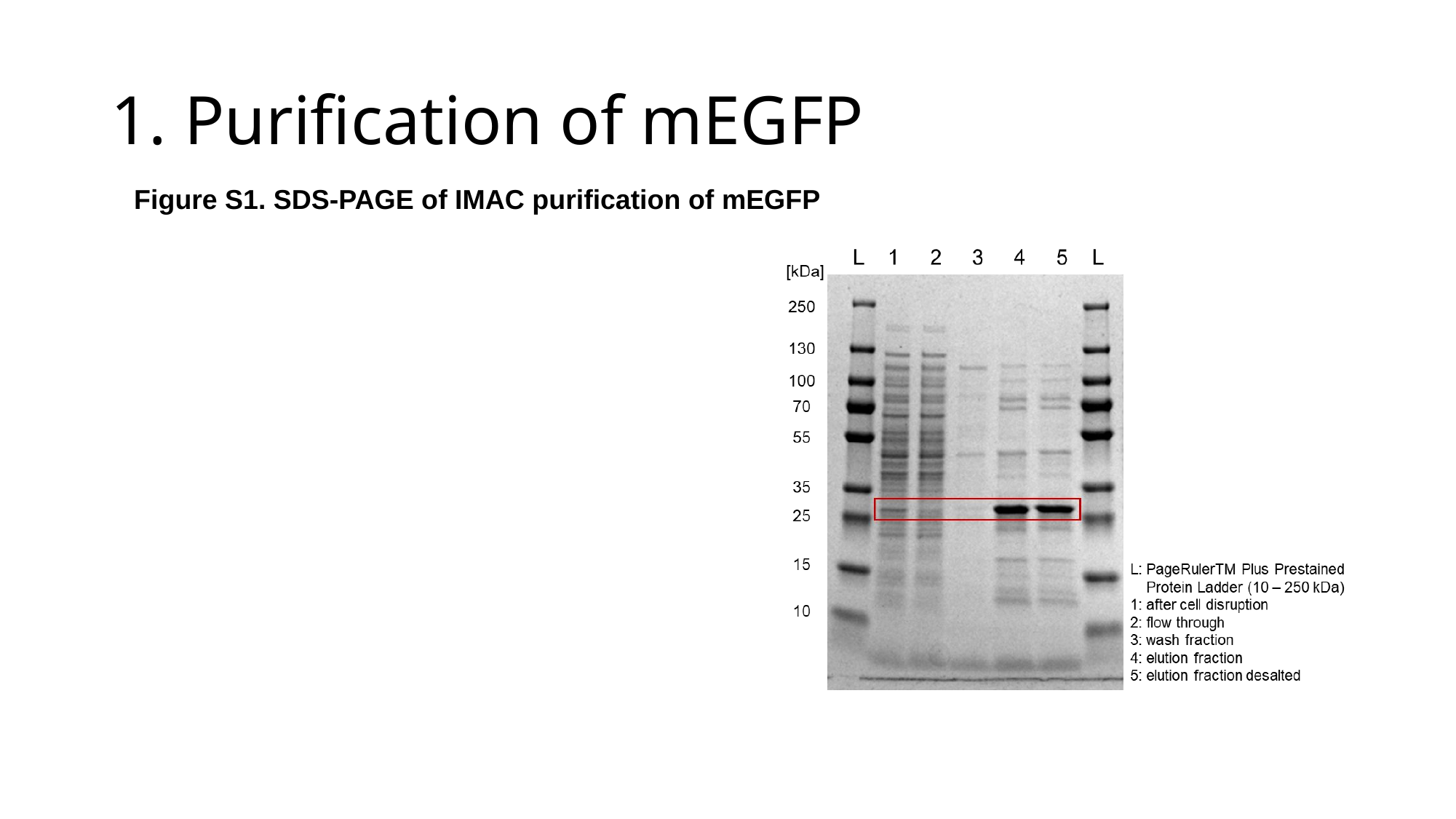

# 1. Purification of mEGFP
Figure S1. SDS-PAGE of IMAC purification of mEGFP

## Slide 3
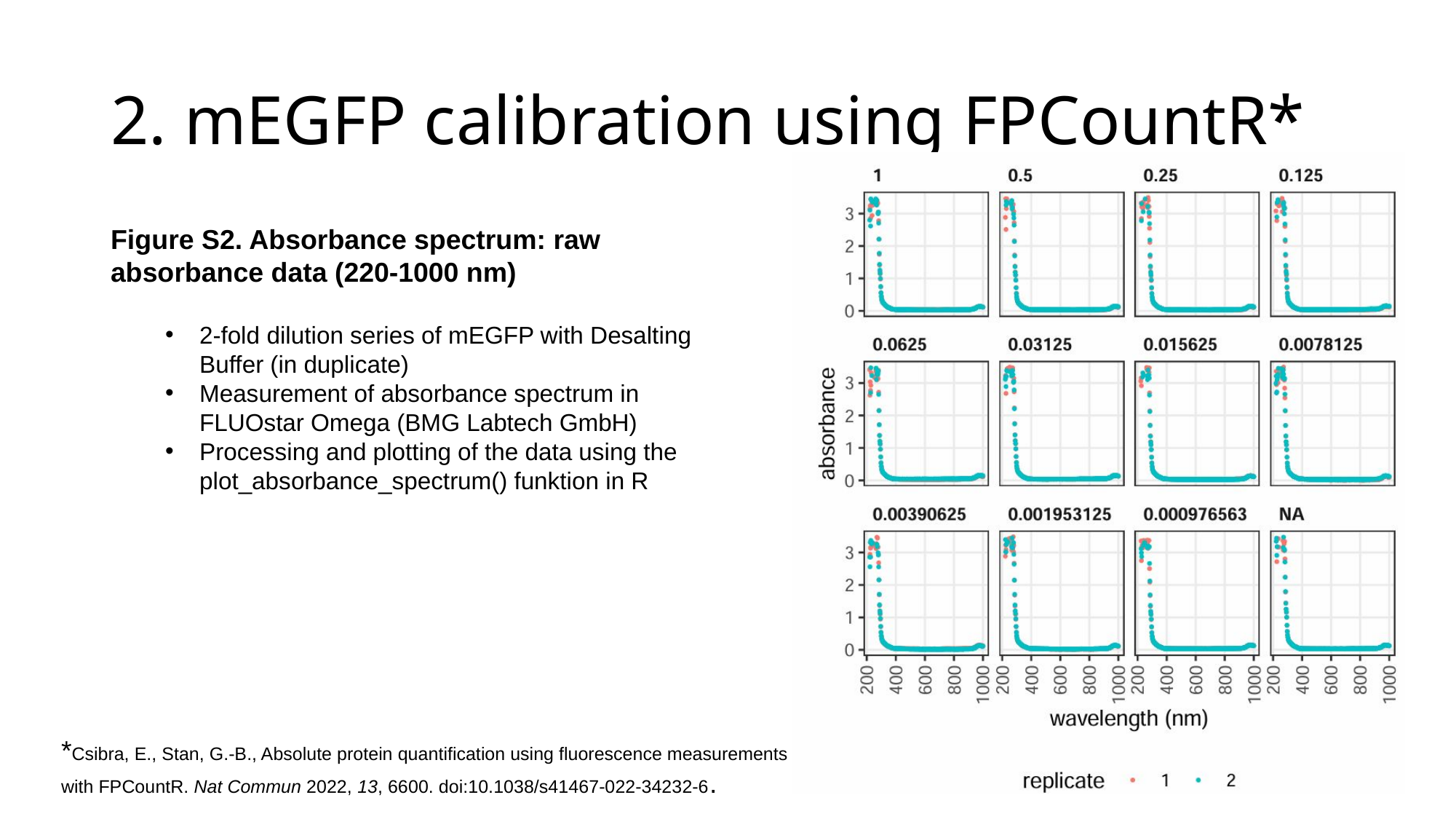

# 2. mEGFP calibration using FPCountR*
Figure S2. Absorbance spectrum: raw absorbance data (220-1000 nm)
2-fold dilution series of mEGFP with Desalting Buffer (in duplicate)
Measurement of absorbance spectrum in FLUOstar Omega (BMG Labtech GmbH)
Processing and plotting of the data using the plot_absorbance_spectrum() funktion in R
*Csibra, E., Stan, G.-B., Absolute protein quantification using fluorescence measurements with FPCountR. Nat Commun 2022, 13, 6600. doi:10.1038/s41467-022-34232-6.

## Slide 4
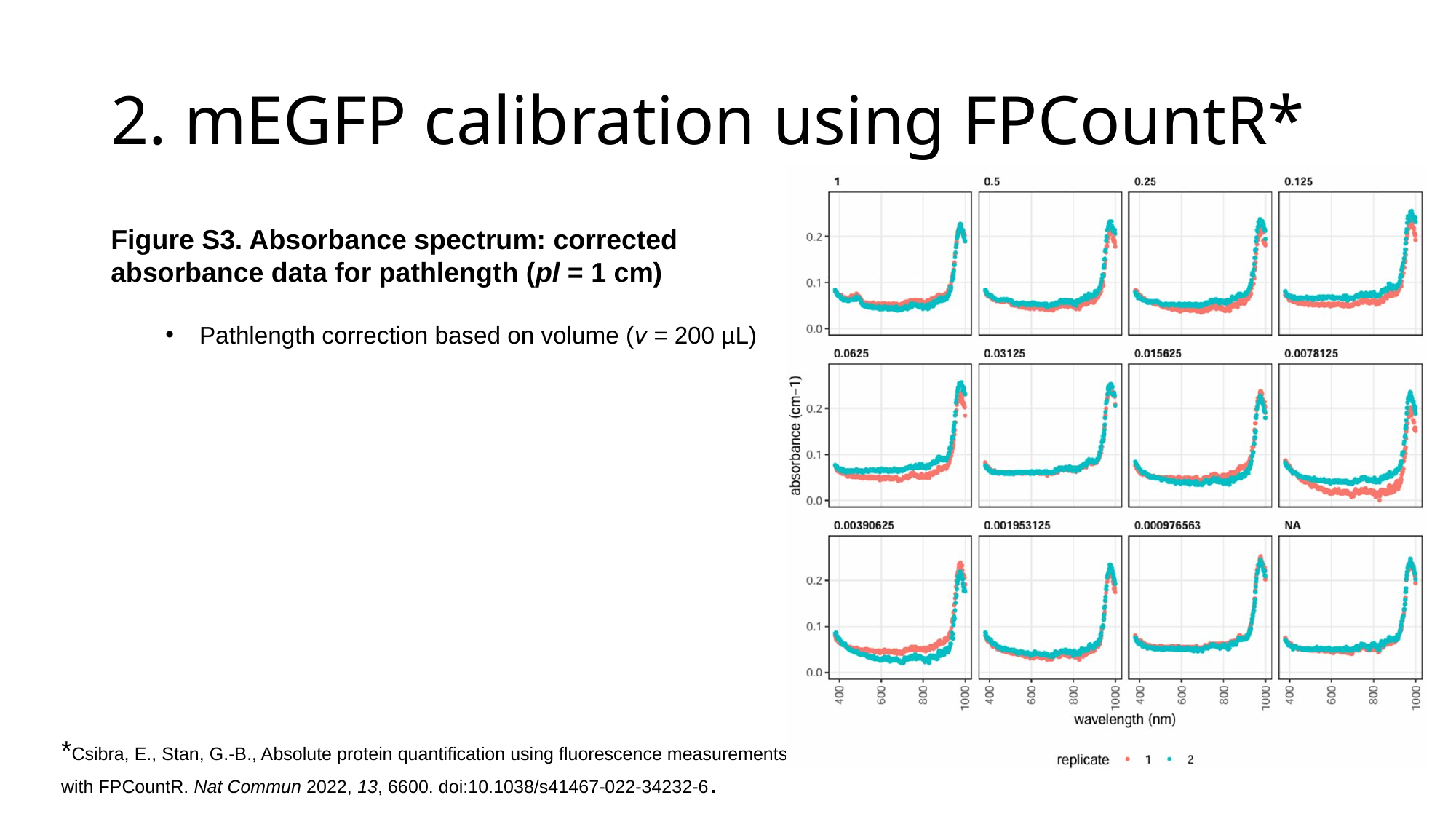

# 2. mEGFP calibration using FPCountR*
Figure S3. Absorbance spectrum: corrected absorbance data for pathlength (pl = 1 cm)
Pathlength correction based on volume (v = 200 µL)
*Csibra, E., Stan, G.-B., Absolute protein quantification using fluorescence measurements with FPCountR. Nat Commun 2022, 13, 6600. doi:10.1038/s41467-022-34232-6.

## Slide 5
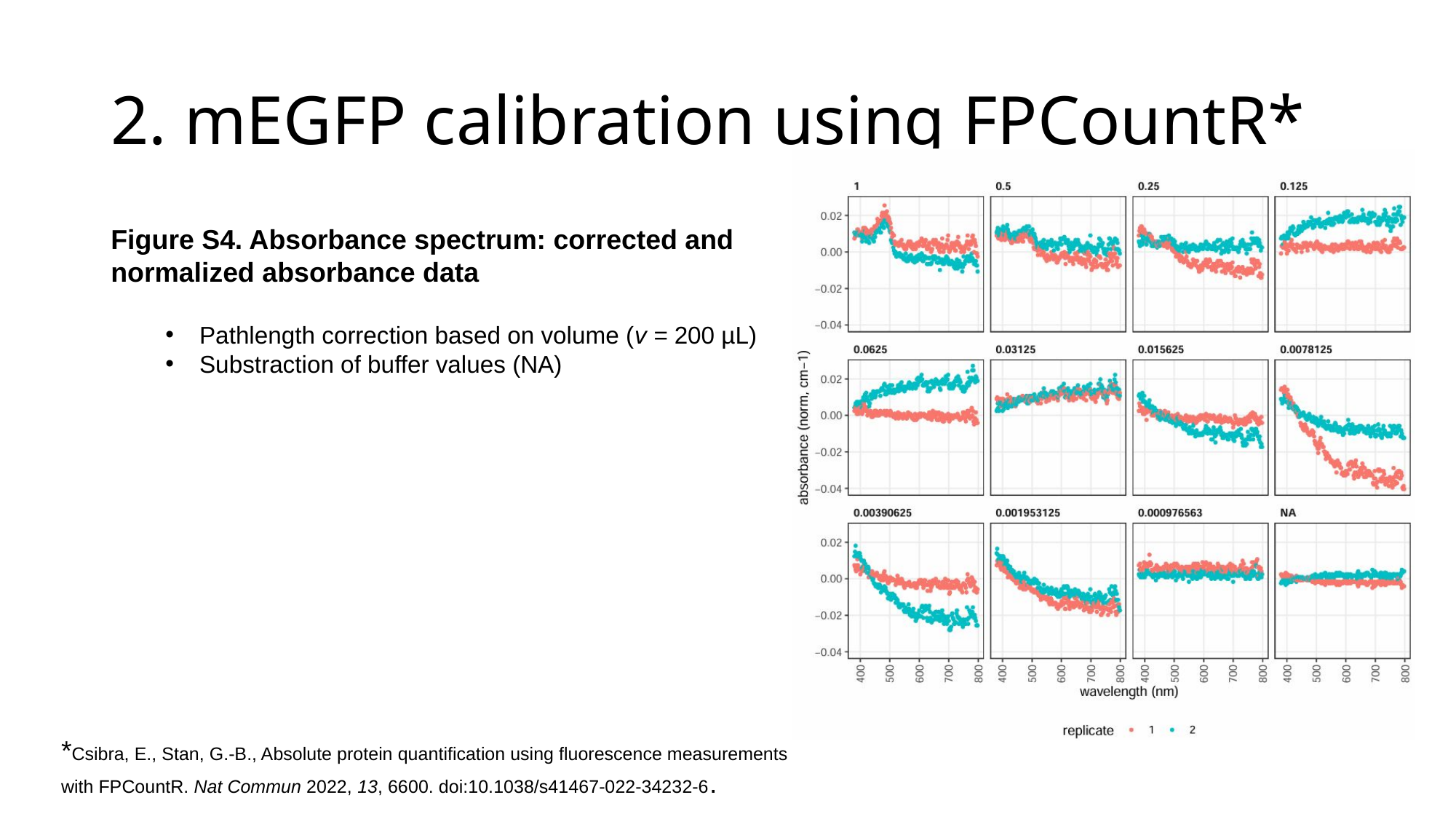

# 2. mEGFP calibration using FPCountR*
Figure S4. Absorbance spectrum: corrected and normalized absorbance data
Pathlength correction based on volume (v = 200 µL)
Substraction of buffer values (NA)
*Csibra, E., Stan, G.-B., Absolute protein quantification using fluorescence measurements with FPCountR. Nat Commun 2022, 13, 6600. doi:10.1038/s41467-022-34232-6.

## Slide 6
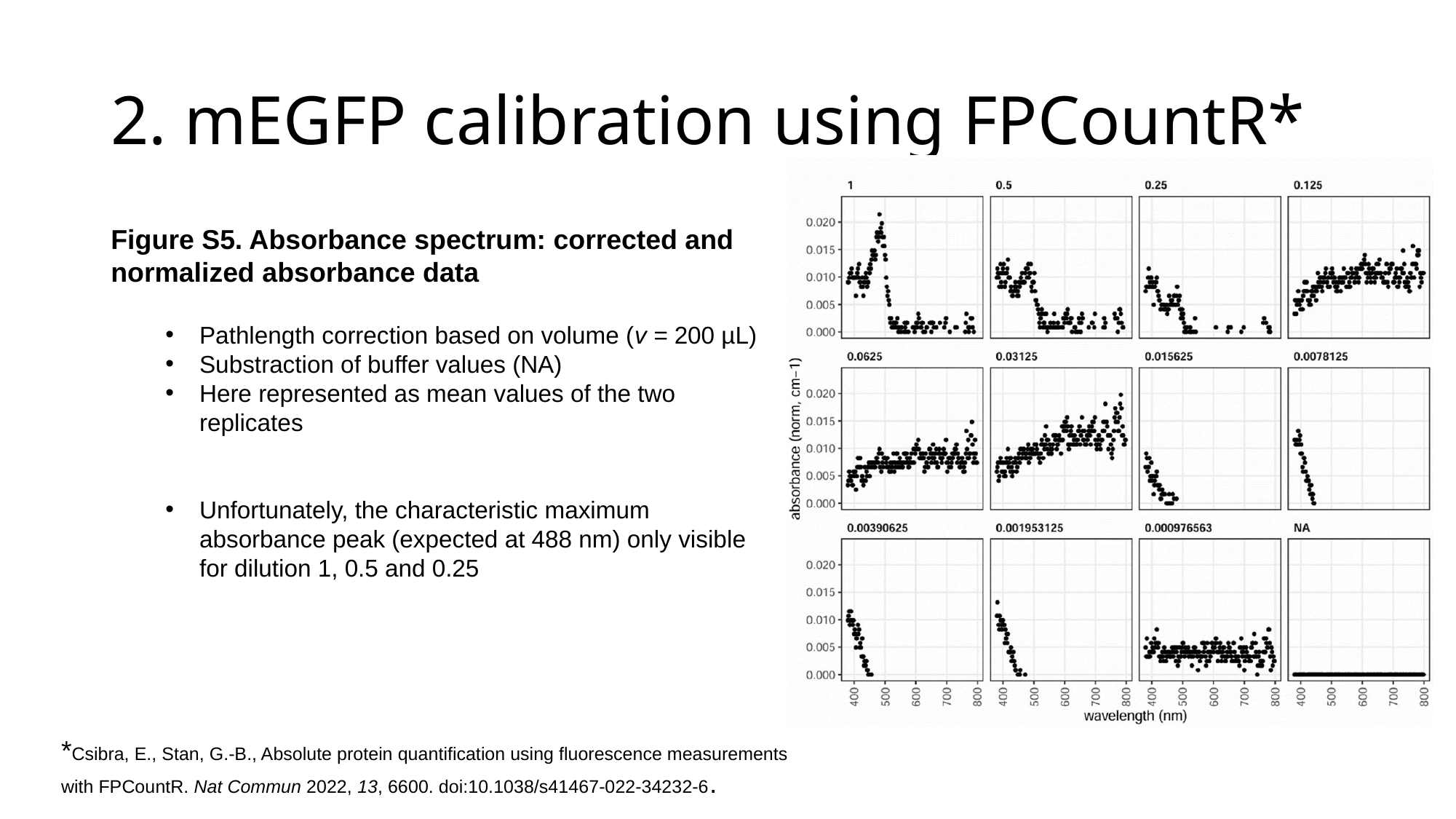

# 2. mEGFP calibration using FPCountR*
Figure S5. Absorbance spectrum: corrected and normalized absorbance data
Pathlength correction based on volume (v = 200 µL)
Substraction of buffer values (NA)
Here represented as mean values of the two replicates
Unfortunately, the characteristic maximum absorbance peak (expected at 488 nm) only visible for dilution 1, 0.5 and 0.25
*Csibra, E., Stan, G.-B., Absolute protein quantification using fluorescence measurements with FPCountR. Nat Commun 2022, 13, 6600. doi:10.1038/s41467-022-34232-6.

## Slide 7
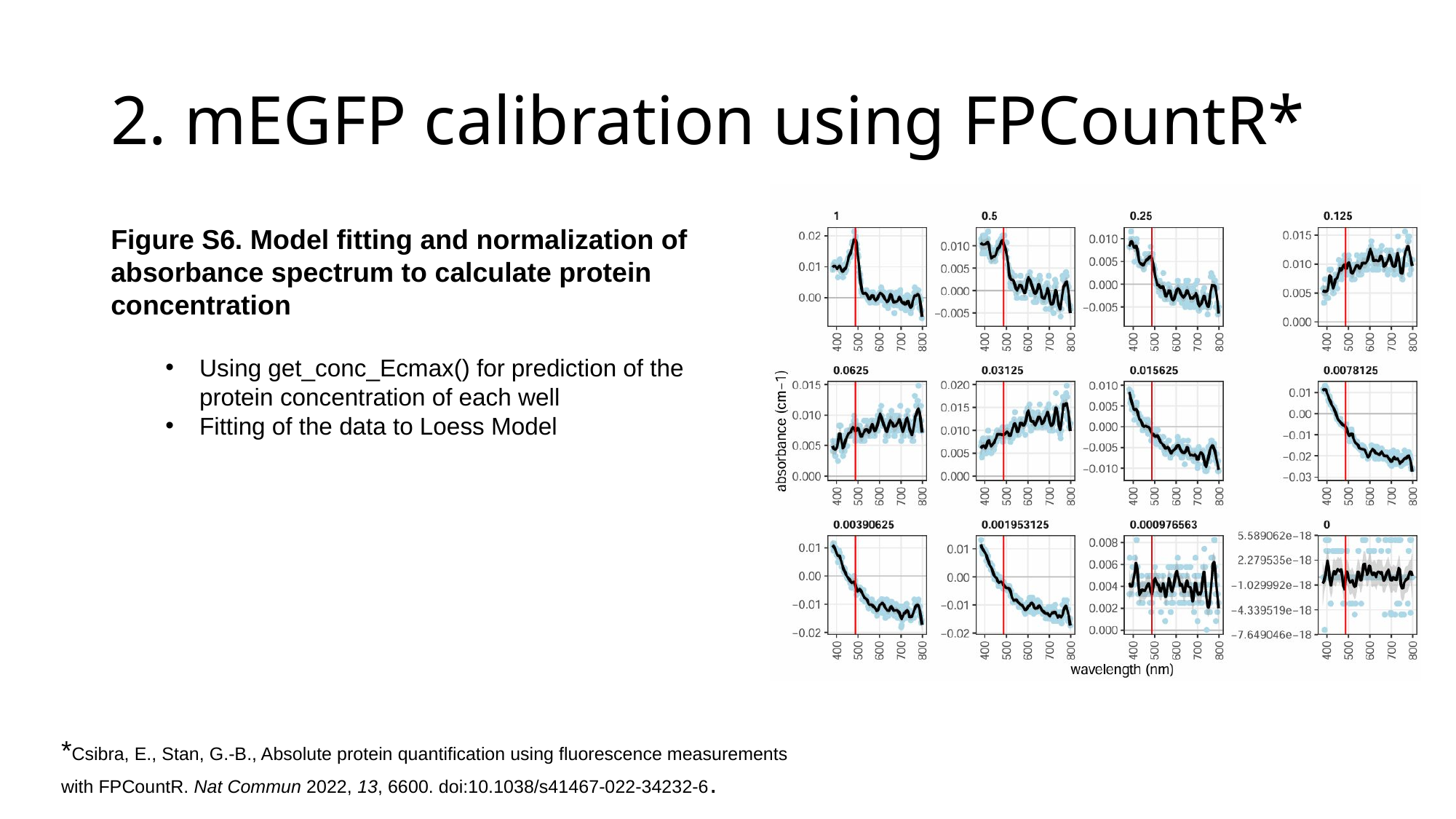

# 2. mEGFP calibration using FPCountR*
Figure S6. Model fitting and normalization of absorbance spectrum to calculate protein concentration
Using get_conc_Ecmax() for prediction of the protein concentration of each well
Fitting of the data to Loess Model
*Csibra, E., Stan, G.-B., Absolute protein quantification using fluorescence measurements with FPCountR. Nat Commun 2022, 13, 6600. doi:10.1038/s41467-022-34232-6.

## Slide 8
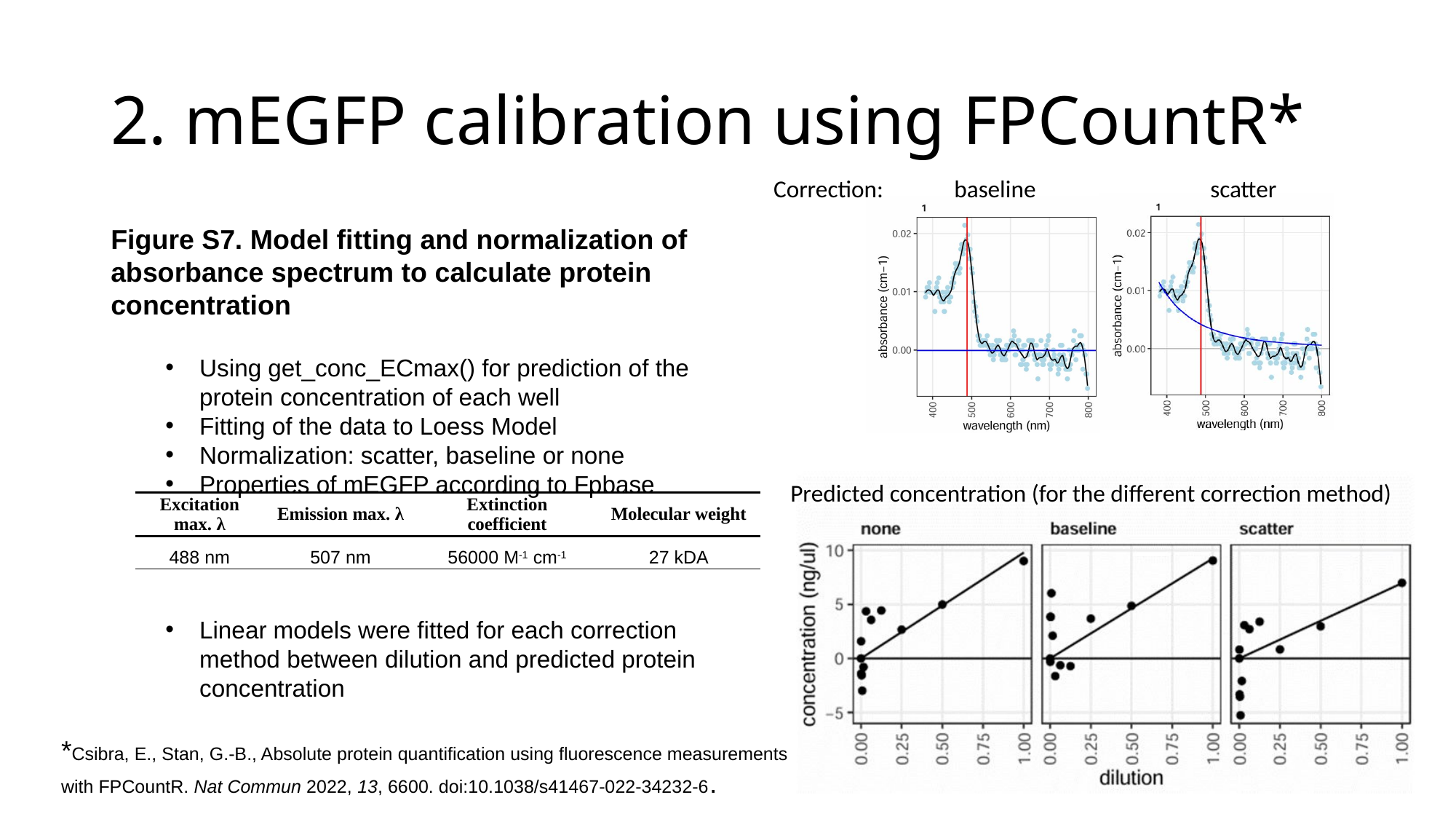

# 2. mEGFP calibration using FPCountR*
Correction: baseline scatter
Figure S7. Model fitting and normalization of absorbance spectrum to calculate protein concentration
Using get_conc_ECmax() for prediction of the protein concentration of each well
Fitting of the data to Loess Model
Normalization: scatter, baseline or none
Properties of mEGFP according to Fpbase
Linear models were fitted for each correction method between dilution and predicted protein concentration
Predicted concentration (for the different correction method)
| Excitation max. λ | Emission max. λ | Extinction coefficient | Molecular weight |
| --- | --- | --- | --- |
| 488 nm | 507 nm | 56000 M-1 cm-1 | 27 kDA |
*Csibra, E., Stan, G.-B., Absolute protein quantification using fluorescence measurements with FPCountR. Nat Commun 2022, 13, 6600. doi:10.1038/s41467-022-34232-6.

## Slide 9
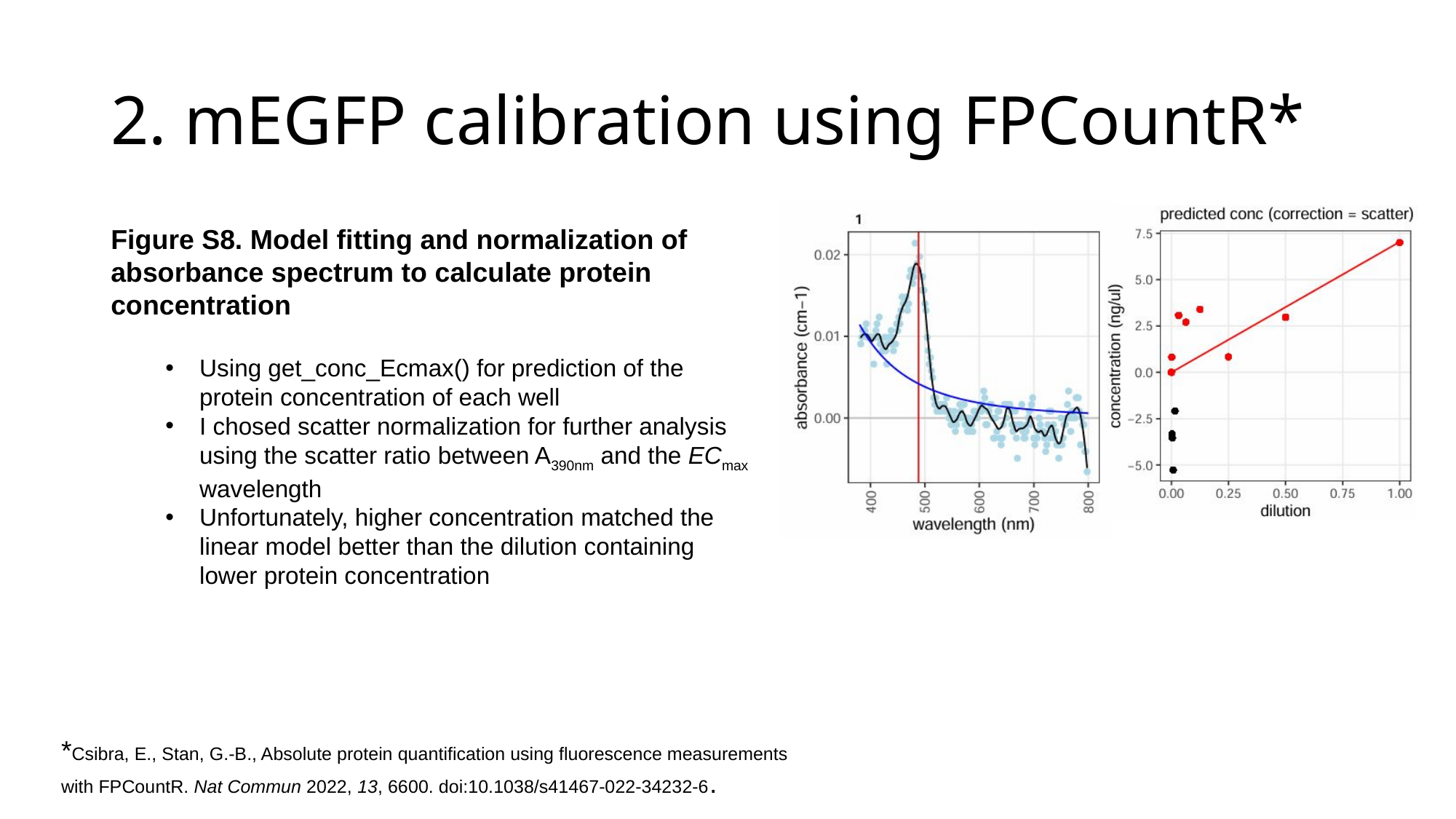

# 2. mEGFP calibration using FPCountR*
Figure S8. Model fitting and normalization of absorbance spectrum to calculate protein concentration
Using get_conc_Ecmax() for prediction of the protein concentration of each well
I chosed scatter normalization for further analysis using the scatter ratio between A390nm and the ECmax wavelength
Unfortunately, higher concentration matched the linear model better than the dilution containing lower protein concentration
*Csibra, E., Stan, G.-B., Absolute protein quantification using fluorescence measurements with FPCountR. Nat Commun 2022, 13, 6600. doi:10.1038/s41467-022-34232-6.

## Slide 10
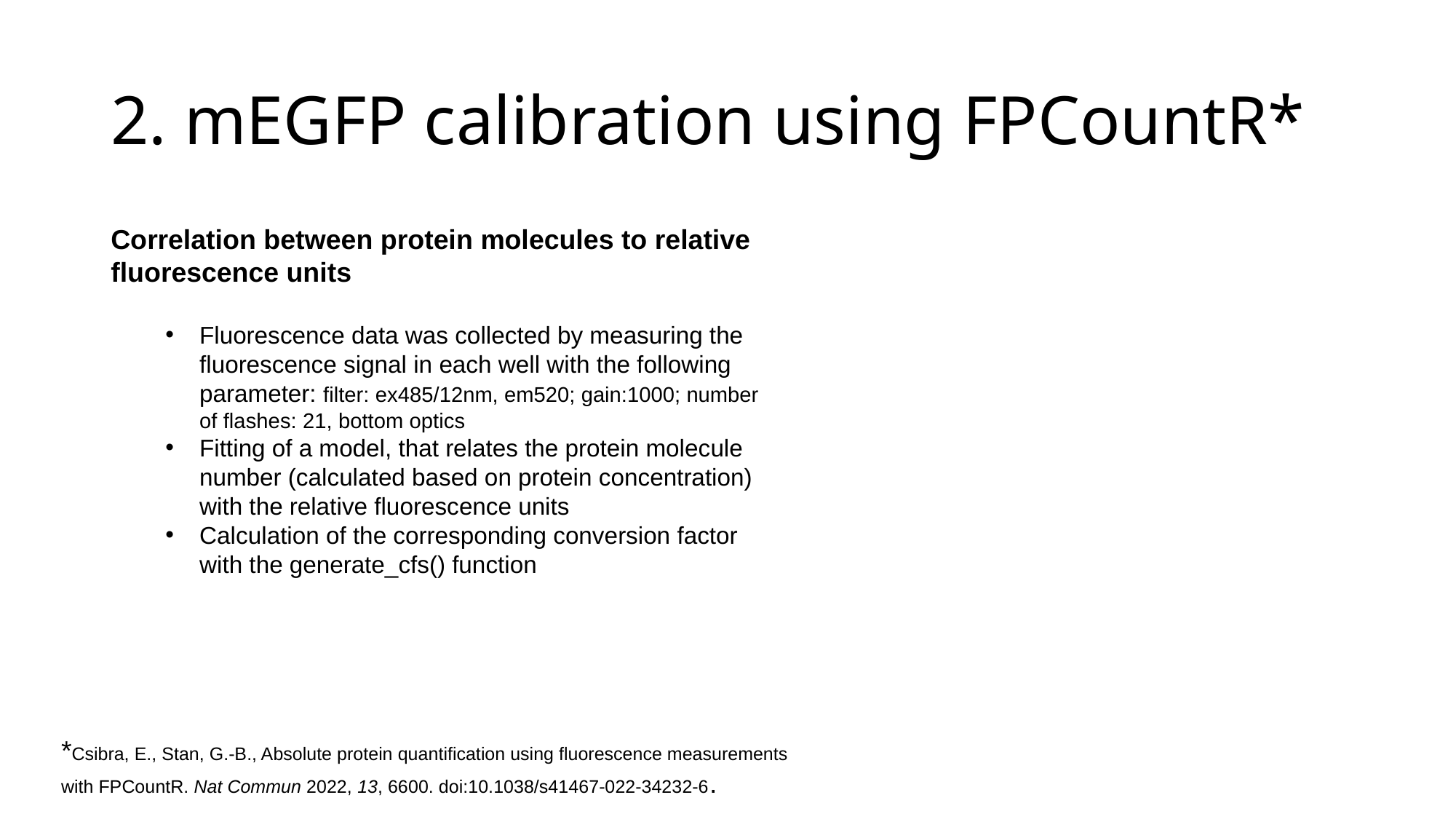

# 2. mEGFP calibration using FPCountR*
Correlation between protein molecules to relative fluorescence units
Fluorescence data was collected by measuring the fluorescence signal in each well with the following parameter: filter: ex485/12nm, em520; gain:1000; number of flashes: 21, bottom optics
Fitting of a model, that relates the protein molecule number (calculated based on protein concentration) with the relative fluorescence units
Calculation of the corresponding conversion factor with the generate_cfs() function
*Csibra, E., Stan, G.-B., Absolute protein quantification using fluorescence measurements with FPCountR. Nat Commun 2022, 13, 6600. doi:10.1038/s41467-022-34232-6.

## Slide 11
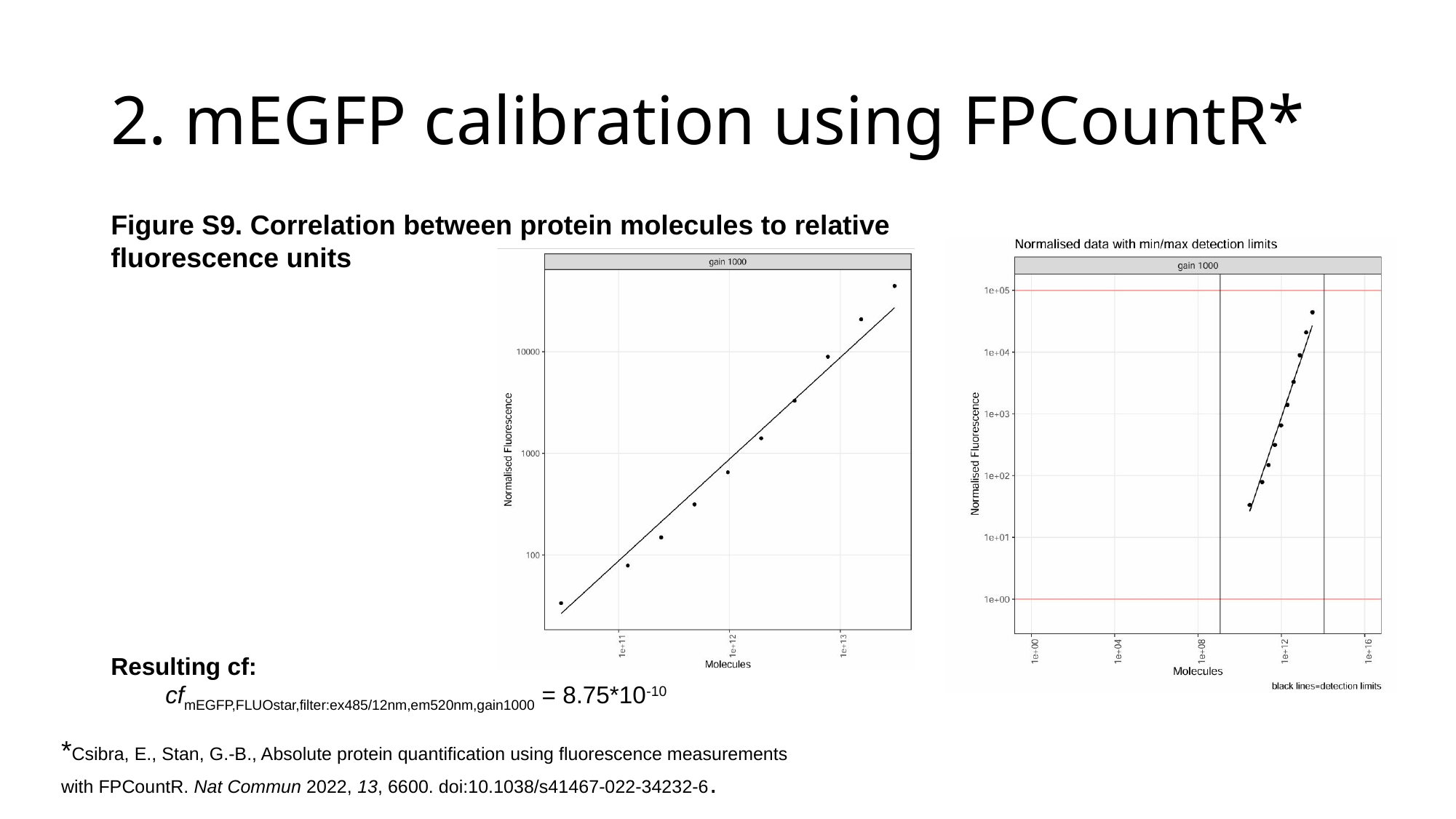

# 2. mEGFP calibration using FPCountR*
Figure S9. Correlation between protein molecules to relative fluorescence units
Resulting cf:
cfmEGFP,FLUOstar,filter:ex485/12nm,em520nm,gain1000 = 8.75*10-10
*Csibra, E., Stan, G.-B., Absolute protein quantification using fluorescence measurements with FPCountR. Nat Commun 2022, 13, 6600. doi:10.1038/s41467-022-34232-6.
