# Supplementary material for: In Vivo Quantification of Surfactin Nonribosomal Peptide Synthetase Complexes in Bacillus subtilis
Source: Microorganisms. 2024 Nov 20;12(11):2381. doi: 10.3390/microorganisms12112381 (PMC11596262; doi:10.3390/microorganisms12112381)
Supplement: Supplementary file 1 [file microorganisms-12-02381-s001.zip › Supplementary File S1.pdf]

Supplementary File S1

In vivo quantification of surfactin nonribosomal peptide synthetase complexes in *Bacillus subtilis*

Maliheh Vahidinasab[1*], Lisa Thewes[1], Bahar Abrishamchi[1], Lars Lilge[1], Susanne Reiße[2], Elvio Henrique Benatto Perino[1] and Rudolf Hausmann[1*]

[1]Department of Bioprocess Engineering (150k), Institute of Food Science and Biotechnology, University of Hohenheim, Fruwirthstrasse 12, 70599 Stuttgart, Germany

[2]Imaging Unit, Core Facility of Hohenheim, Emil-Wolff-Strasse 12, 70599 Stuttgart, Germany

* Correspondence: vahidin@uni-hohenheim.de, rudolf.hausmann@uni-hohenheim.de

Table S1. Oligonucleotides used in this study

Order	Oligonucleotide name	Sequence (5' → 3')	Application	
1	Vector_*srfAA*_F	ACGAGATGCTGGGATCCTCTAGATTTCTATAGTGTCACCT	Amplification of pJOE6743.1 backbone for using as the vector	Construction of pMAV22
2	Vector_*srfAA*_R	TCCAGTAAAATCCGTCCCTATAGTGAGTCGTATTAGGCATGC		
3	up_*srfAA*_F	CTCACTATAGGGACGGATTTTACTGGAGGATTTCgcc	Amplification of end of *srfAA* gene with excluding the stop codon	
4	up-*srfAA*-R	CTTCTCCTTTACTAACCATgaaaatttccattaatttatccagctcatccag		
5	eGFP-*srfAA*-F	ggataaattaatggaaattttCATGGTTAGTAAAGGAGAAGAACTTTTCACTG	Amplification of *mEGFP*	
6	eGFP-*srfAA*-R	ctcatatgccacctctTTATTTGTATAGTTCATCCATGCCAAGTGTAATC		
7	down-*srfAA*_F	GATGAACTATACAAATAAagaggtggcatatgagcaaaaaatc	Amplification of downstream region of *srfAA*	
8	down_*srfAA*_R	CTAGAGGATCCCAGCATCTCGTTCAACTTTCACcc		
9	Vector_*srfAB*_F	tcTATAGTGAGTCGCCCTATAGTGAGTCGTATTAGGCATGC	Amplification of pJOE6743.1 backbone for using as the vector	Construction of pMAV23
10	Vector_*srfAB*_R	TCCAGTAAAATCCGTCCCTATAGTGAGTCGTATTAGGCATGC		
11	up_*srfAB*_F	CGACTCACTATAGGGCGACTCACTATAgactttgcttcaggctacatgcag	Amplification of end of *srfAB*gene with excluding the stop codon	
12	up-*srfAB*-R	CAGTGAAAAGTTCTTCTCCTTTACTAACCATttttaaattctcctcaagcatgtcaaagatatctcc		
13	eGFP-*srfAB*-F	gagatatctttgacatgcttgaggagaatttaaaaATGGTTAGTAAAGGAGAAGAACTTTTCACTG	Amplification of *mEGFP*	
14	eGFP-*srfAB*-R	gccttctgtaattcccttgcgttTTATTTGTATAGTTCATCCATGCCAAGTGTAATCC		
15	down-*srfAB*_F	GATTACACTTGGCATGGATGAACTATACAAATAAacgcaagggaattacagaagg	Amplification of downstream region of *srfAB*	
16	down_*srfAB*_R	TAGAGGATCCCCGGccgtactcaaagtggtatgctgc		

Order	Oligonucleotide name	Sequence (5' → 3')	Application	
17	Vector_*srfAC*_F	tgtattgctgtttacgtttgGGGATCCTCTAGATTTCTATAGTGTCACC	Amplification of pJOE6743.1 backbone for using as the vector	Construction of pMAV24
18	Vector_*srfAC*_R	gctcatccaagaaggtaaagTCCCTATAGTGAGTCGTATTAGGCATGC		
19	up_*srfAC*_F	CTATAGGGActttaccttcttggatgagcttcct	Amplification of end of *srfAB*gene with excluding the stop codon	
20	up-*srfAC*-R	CTCCTTTACTAACCATtgaaaccgttacggtttgtgtattaagaaattc		
21	eGFP-*srfAC*-F	ccgtaacggtttcaATGGTTAGTAAAGGAGAAGAACTTTTCACTG	Amplification of *mEGFP*	
22	eGFP-*srfAC*-R	catcacttcatTTATTTGTATAGTTCATCCATGCCAAGTGTAATCC		
23	down-*srfAC*_F	GGCATGGATGAACTATACAAATAAatgaagtgatgaaaggaggagacagc	Amplification of downstream region of *srfAC*	
24	down_*srfAC*_R	GGATCCCcaaacgtaaacagcaatacaaaccatttcatcc		
25	Vector_*srfAD*_F	cattcggctgGGGGATCCTCTAGATTTCTATAGTGTCACC	Amplification of pJOE6743.1 backbone for using as the vector	Construction of pMAV25
26	Vector_*srfAD*_R	AGTAAAAGGCATGTCCCTATAGTGAGTCGTATTAGGCATGC		
27	up_*srfAD*_F	CGACTCACTATAGGGACATGCCTTTTACTCATACTACGTCAACc	Amplification of end of *srfAB*gene with excluding the stop codon	
28	up-*srfAD*-R	CTTCTCCTTTACTAACCATcggttgaatgatcggatgctg		
29	eGFP-*srfAD*-F	gatcattcaaccgATGGTTAGTAAAGGAGAAGAACTTTTCACTG	Amplification of *mEGFP*	
30	eGFP-*srfAD*-R	ctgtccgcttttgaTTATTTGTATAGTTCATCCATGCCAAGTGTAATCC		
31	down-*srfAD*_F	GGATGAACTATACAAATAAtcaaaagcggacagcttcgg	Amplification of downstream region of *srfAD*	
32	down_*srfAD*_R	GAGGATCCCCcagccgaatgaaaaataagatggatagcat		
33	mEGFP expression_F	gtttaactttaagaaggagatatacCCATGGTTAGTAAAGGAGAAGAACTTTTCACTG	Amplification of *mEGFP*	Construction of pMAV35
34	mEGFP expression_R	gtggtggtgctcgagtgcTTTGTATAGTTCATCCATGCCAAGTGTAATC		

Table S2. Strains and plasmids used in this study.

Strain or plasmid	Genotype or description	Reference
**Strains**		
Escherichia coli		
**DH5α**	Δ*(argF-lac)169*,φ80d*lacZ58*(M15),Δ*phoA8*, *glnX44*(AS), *deoR481*, *rfbC1*, *gyrA96*(NalR), *recA1*, *endA1*, *thiE1* and *hsdR17*	(Song *et al.*, 2015)
**BL21 (DE3) Gold**	*F hsdSgal DE3*	
Bacillus subtilis		
**3NA**	*spo0A3*;	(Reuß *et al.*, 2016)
**BMV9**	*spo0A3*; Δ*manPA*; *sfp*+;	(Vahidinasab *et al.*, 2020)
**BMV25**	*spo0A3*; Δ*manPA*; *sfp*+ ; *srfAA-megfp* *mEGFP is Chromosomally integrated at the end of srfAA before the stop codon*	This study
**BMV26**	*spo0A3*; Δ*manPA*; *sfp*+ ; *srfAB-megfp* *mEGFP is Chromosomally integrated at the end of srfAB before the stop codon*	This study
**BMV27**	*spo0A3*; Δ*manPA*; *sfp*+ ; *srfAC-megfp* *mEGFP is Chromosomally integrated at the end of srfAC before the stop codon*	This study
**BMV28**	*spo0A3*; Δ*manPA*; *sfp*+ ; *srfAD-megfp* *mEGFP is Chromosomally integrated at the end of srfAD before the stop codon*	This study
**Plasmids**		
**pJOE6743.1**	*ori*$_{\text{pUC18}}$,*bla, spc*, *manP*, *ter-'lacI-lacZα-ter*	(Wenzel and Altenbuchner, 2015)
**pET-28a**	Expression plasmid, *ori, kanR, lacI, his(7), tev site*	#20061, Addgene, Watertown, USA (Denisov *et al.*, 2004)
**pMAV22**	pJOE6743.1 containing *srfAA´-megfp-´srfAB* (1000 bp up- and downstream of the C-terminal end of *srfAA* gene were used as flanking regions of the *megfp* gene)	This study
**pMAV23**	pJOE6743.1 containing *srfAB´-megfp-´srfAC* (1000 bp up- and downstream of the C-terminal end of *srfAB* gene were used as flanking regions of the *megfp* gene)	This study
**pMAV24**	pJOE6743.1 containing *srfAC´-megfp-´srfAD* (1000 bp up- and downstream of the C-terminal end of *srfAC* gene were used as flanking regions of the *megfp* gene)	This study
**pMAV25**	pJOE6743.1 containing *srfAD´-megfp-´ycxA* (1000 bp up- and downstream of the C-terminal end of *srfAD* gene were used as flanking regions of the *megfp* gene)	This study
**pMAV35**	his-mEGFP expression plasmid using pET28a as expression vector	This study

References

Denisov, I.G., Grinkova, Y.V., Lazarides, A.A., and Sligar, S.G. (2004) Directed self-assembly of monodisperse phospholipid bilayer Nanodiscs with controlled size. *Journal of the American Chemical Society* **126** (11): 3477–3487.

Reuß, D.R., Thürmer, A., Daniel, R., Quax, W.J., and Stülke, J. (2016) Complete genome sequence of Bacillus subtilis subsp. subtilis strainΔ 6. *Genome announcements* **4** (4): 10.1128/genomea. 00759-16.

Song, Y., Lee, B.-R., Cho, S., Cho, Y.-B., Kim, S.-W., Kang, T.J., *et al.* (2015) Determination of single nucleotide variants in Escherichia coli DH5α by using short-read sequencing. *FEMS Microbiology Letters* **362** (11): fnv073.

Vahidinasab, M., Lilge, L., Reinfurt, A., Pfannstiel, J., Henkel, M., Morabbi Heravi, K., and Hausmann, R. (2020) Construction and description of a constitutive plipastatin mono-producing Bacillus subtilis. *Microbial Cell Factories* **19:** 1–12.

Wenzel, M., and Altenbuchner, J. (2015) Development of a markerless gene deletion system for Bacillus subtilis based on the mannose phosphoenolpyruvate-dependent phosphotransferase system. *Microbiology* **161** (10): 1942–1949.
